# Changes in temperature in preheated crystalloids at ambient temperatures relevant to a prehospital setting: an experimental simulation study with the application of prehospital treatment of trauma patients suffering from accidental hypothermia

**DOI:** 10.1186/s12873-024-00969-0

**Published:** 2024-04-12

**Authors:** Emil Jensen, Helena Rentzhog, Johan Herlitz, Christer Axelsson, Peter Lundgren

**Affiliations:** 1 University of Borås, Borås, Sweden; 2 Centre for Prehospital Research, University of Borås, Borås, Sweden; 3 Department of Prehospital Emergency Care, Sahlgrenska University Hospital, Gothenburg, Sweden; 4 Institute of Medicine, University of Gothenburg, Gothenburg, Sweden

**Keywords:** Accidental hypothermia, Advanced trauma life support care, Resuscitation

## Abstract

**Background:**

Accidental hypothermia is common in all trauma patients and contributes to the lethal diamond, increasing both morbidity and mortality. In hypotensive shock, fluid resuscitation is recommended using fluids with a temperature of 37–42°, as fluid temperature can decrease the patient’s body temperature. In Sweden, virtually all prehospital services use preheated fluids. The aim of the present study was to investigate how the temperature of preheated infusion fluids is affected by the ambient temperatures and flow rates relevant for prehospital emergency care.

**Methods:**

In this experimental simulation study, temperature changes in crystalloids preheated to 39 °C were evaluated. The fluid temperature changes were measured both in the infusion bag and at the patient end of the infusion system. Measurements were conducted in conditions relevant to prehospital emergency care, with ambient temperatures varying between − 4 and 28 °C and flow rates of 1000 ml/h and 6000 ml/h, through an uninsulated infusion set at a length of 175 cm.

**Results:**

The flow rate and ambient temperature affected the temperature in the infusion fluid both in the infusion bag and at the patient end of the system. A lower ambient temperature and lower flow rate were both associated with a greater temperature loss in the infusion fluid.

**Conclusion:**

This study shows that both a high infusion rate and a high ambient temperature are needed if an infusion fluid preheated to 39 °C is to remain above 37 °C when it reaches the patient using a 175-cm-long uninsulated infusion set. It is apparent that the lower the ambient temperature, the higher the flow rate needs to be to limit temperature loss of the fluid.

## Background

Accidental hypothermia, together with coagulopathy and acidosis, constitutes the lethal triad. Patients presenting with all three components have a very high mortality rate [1, 2]. Hypocalcemia has recently been added, making the lethal triad a lethal diamond [3, 4]. In trauma, mortality risk starts to increase at a body temperature of 36 °C [5–7] and continues to increase with decreases in body temperature [8] to 32 °C, where it remains at approximately 40% even if the body temperature decreases even further [9].

Among patients arriving at a trauma center, it has been reported that a body temperature of < 35 °C is found in up to 23% of cases [5–7, 10–12]. These patients have a hospital stay of two to three days longer than trauma patients who do not present with hypothermia [6, 7]. Among trauma patients who were admitted to an intensive care unit (ICU), a body temperature below 35 °C was reported in 37% of cases. These patients had an increased risk of death during the subsequent 24 h as well as during the subsequent 28 days [13].

Severe trauma inhibits the body’s temperature regulation and is the strongest risk factor for accidental hypothermia on arrival at a trauma center [6, 7, 10–12]. In severe trauma, one study reported that 73% of patients had a body temperature of < 35 °C at the first measurement by the ambulance after arriving on the scene. The temperature continued to decrease in 91% of the patients during prehospital care [14]. Another study reported that approximately 30% of trauma patients transported by ambulance to a trauma facility already had a body temperature < 35 °C at the scene of injury, regardless of the severity of the accident [15].

Reported risk factors for accidental hypothermia in trauma patients include the volume of prehospital fluid therapy [10, 11] and the temperature in the prehospital fluid administered [12]. In hypotensive shock, the aim of fluid therapy is to achieve acceptable tissue perfusion to vital organs, since hypoperfusion increases the risk of acidosis [16]. Hypotensive fluid resuscitation increases survival compared with “no fluid therapy” [17].

In fluid resuscitation, warm fluids are recommended with a specified temperature of between 37 and 42 °C [18–22]. Perioperative fluid infusions have been shown to decrease body temperature, and fluids are often either preheated or heated through an inline infusion warmer. Body temperature has been shown to decrease more among patients who received a room-temperature infusion than among those who received a preheated infusion [23, 24].

In Sweden, preheated fluids are used in virtually all ambulance services. Fewer than half of all ambulances carry equipment to insulate the infusion bag and set, and barely any use an in-line heater [25]. The infusion fluids are affected by heat loss in both the infusion bag [26] and the infusion set [27].

The aim of the present study was to investigate how preheated infusion fluids are affected by the ambient temperature both in their packaging and in the infusion set at different ambient temperatures and flow rates relevant for prehospital emergency care.

## Methods

### Design

The study had an experimental design, and a descriptive approach was applied to changes in preheated crystalloids. The study was divided into two parts. Part one measured temperature changes in the infusion bag, hanging freely at different ambient temperatures, at set time intervals. Part two measured the temperatures during an infusion, in the infusion bag, and 5 cm from the patient end of the infusion line at different infusion flow rates and ambient temperatures.

### Settings

The trials were conducted during the second and third quarters of 2022.

The experiments were conducted indoors in a home environment with a thermostat-controlled room temperature at temperatures of 16.5, 22, and 28 °C. For experiments at − 4 °C, a standard household freezer was used. The ambient temperature in the vicinity of the bag was measured with a thermometer to identify variations in ambient temperature (± 0.2 °C).

The ambient temperatures were chosen to represent different prehospital scenarios, and the trials were conducted at ambient temperatures of 28, 22, 16.5, and − 4 °C. An ambient temperature of 28 °C is recommended when treating patients suffering from accidental hypothermia [18], 22 °C can be considered a normal indoor temperature in Sweden [28], and 16.5 °C was chosen to not exceed 17 °C, as this has been identified as a significant ambient temperature for the risk of accidental hypothermia [14]. Finally, − 4 °C is the average temperature for the middle of Sweden in January [29].

The infusion bags were preheated to 39 °C (+–0.2 °C) based on the highest possible temperature according to the specification (37 °C (+–2 °C)) of a type of infusion warmer commonly used in Swedish ambulances [30].

Throughout all the trials, the bags were hanging freely and did not come into contact with any walls or furniture. No insulation was used.

The infusion flow rate for a bolus dose during fluid resuscitation was estimated at approximately 6000 ml/h. To measure the difference between different flow rates, 1000 ml/h was chosen as a comparison. These rates were chosen from our experiences as specialist nurses in prehospital emergency care and after discussion with colleagues within the emergency services. In this study, these two flow rates are referred to as high and low flow rates, respectively.

Before data collection commenced, a control measurement of infusion flow rates was carried out by inserting the temperature probe through different peripheral vein catheters (PVCs) with the purpose of identifying suitable PVCs to achieve the desired infusion flow rates.

### Material

The infusion bags consisted of 1000 ml of Ringer’s acetate (manufacturer: Baxter Viaflo) and were made of soft plastic. The catheter of the intravenous infusion set had a length of 175 cm, and two different PVCs were used. The inner diameters of the two PVCs used were 2.0 mm (14 Gauge, Orange) and 1.5 mm (17 Gauge, White). The temperature measurements were performed with a Testo Thermometer (www.testo.com), model 175 T3 Logger, with a temperature probe measuring the fluid temperature within the infusion bag and/or the infusion line. The probe used for the infusion line had a diameter of 1 mm. Times were measured with a standard stopwatch application on a smartphone.

### Data collection

In part one, the bags were hung up at the selected ambient temperature, and a temperature probe was inserted with a cannula through the injection port and placed in the middle of the bag. To even out the temperature changes in the infusion fluid, the bag was shaken before each measurement. Measurements were conducted every fifth minute for an hour by the authors of the study via the thermometer display. Each trial consisted of two bags, and three trials were conducted for a total of six bags at each ambient temperature.

In part two, an infusion was set with one of the two PVCs connected to the infusion bag. One temperature probe was inserted through a hole in the top of the bag that measured the fluid temperature within the infusion bag adjacent to the infusion set inlet, and one temperature probe was inserted approximately 5 cm into the infusion set catheter through the tip of the PVC. The flow rate regulator was opened to the maximum, and the entire volume of the bag was infused into a transparent container graded every 100 ml. The time was manually noted for every 100 ml, and temperatures were automatically recorded every 10th second by the thermometer. The authors then transcribed the temperatures from the time for each 100 ml from the recorded data. Since the experiment using the household freezer made it impossible to note the time for each 100 ml, in this simulation, the measurement was stopped when the temperature drastically decreased, since this meant that the bag was empty. The time was noted, and an average infusion rate was calculated by dividing the total time by ten. This was used as an approximate time for each 100 ml.

### Data analysis

The data were registered in IBM® SPSS®, which was used for the compilation of descriptive statistics and to create figures. The measurements were compiled as figures, and mean flow rates were calculated for 16.5, 22, and 28 °C individually and collectively.

## Results

### Part 1

**Fig. 1:**
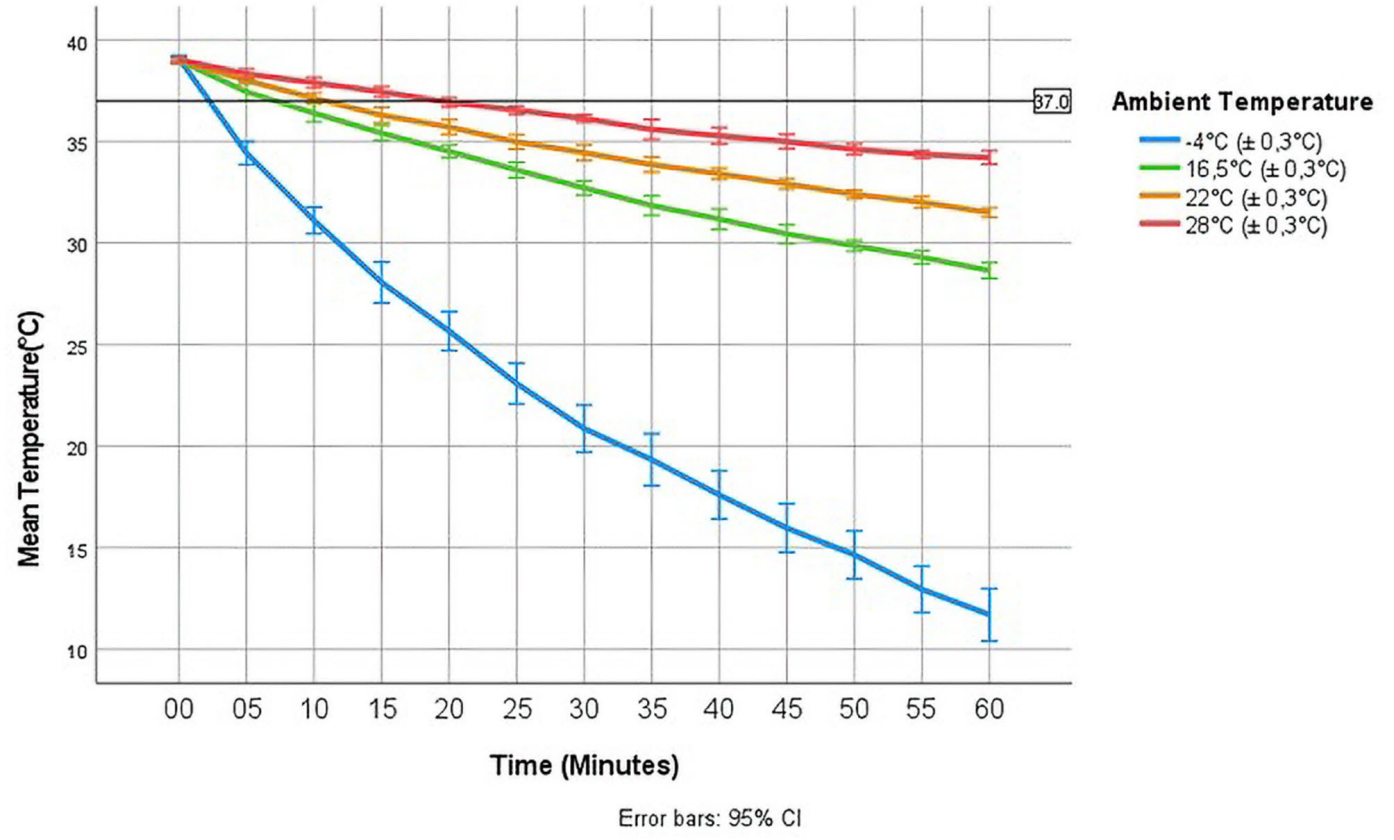
Changes in fluid temperature over time in infusion bags preheated to 39 °C at different ambient temperatures

The temperature in the infusion bag was affected by the ambient temperature, where a colder ambient temperature resulted in a more extensive loss of heat (Fig. 1). Greater differences between fluid and ambient temperatures resulted in greater changes in the fluid temperature.

Infusion fluid preheated to 39 °C reached 37 °C after approximately 20 min at an ambient temperature of 28 °C. If the ambient temperature was 22 °C, the corresponding time was reduced to approximately 10 min; if it was 16.5 °C, the time was further reduced to approximately 7.5 min; and finally, if the ambient temperature was set at − 4 °C, the time was even further reduced to 2.5 min.

### Part 2

The flow rate was higher at the beginning than at the end of the infusion (Table 1). The estimated flow rate at an ambient temperature of − 4 °C was, per 100 ml, 8654 ml/hour for high flow and 1565 ml/hour for low flow.

**Table 1:** Avg. flow rate in ml/h (SD) at different ambient temperatures

Readings	16.5 °C		22 °C		28 °C		All	
	High Flow	Low Flow	High Flow	Low Flow	High Flow	Low Flow	High Flow	Low Flow
100–1000 ml	5954 (351)	982 (139)	5995 (364)	954 (83)	6108 (325)	1072 (161)	6019 (350)	1003 (140)
100–400 ml	6268 (227)	1081 (61)	6326 (266)	1033 (53)	6418 (250)	1198 (57)	6448 (249)	1104 (90)
400–700 ml	5986 (136)	1009 (46)	5993 (89)	951 (36)	6034 (81)	1082 (64)	6005 (104)	1014 (73)
700–1000 ml	5624 (205)	856 (133)	5660 (192)	877 (43)	5851 (183)	941 (167)	5712 (213)	891 (127)

**Fig. 2:**
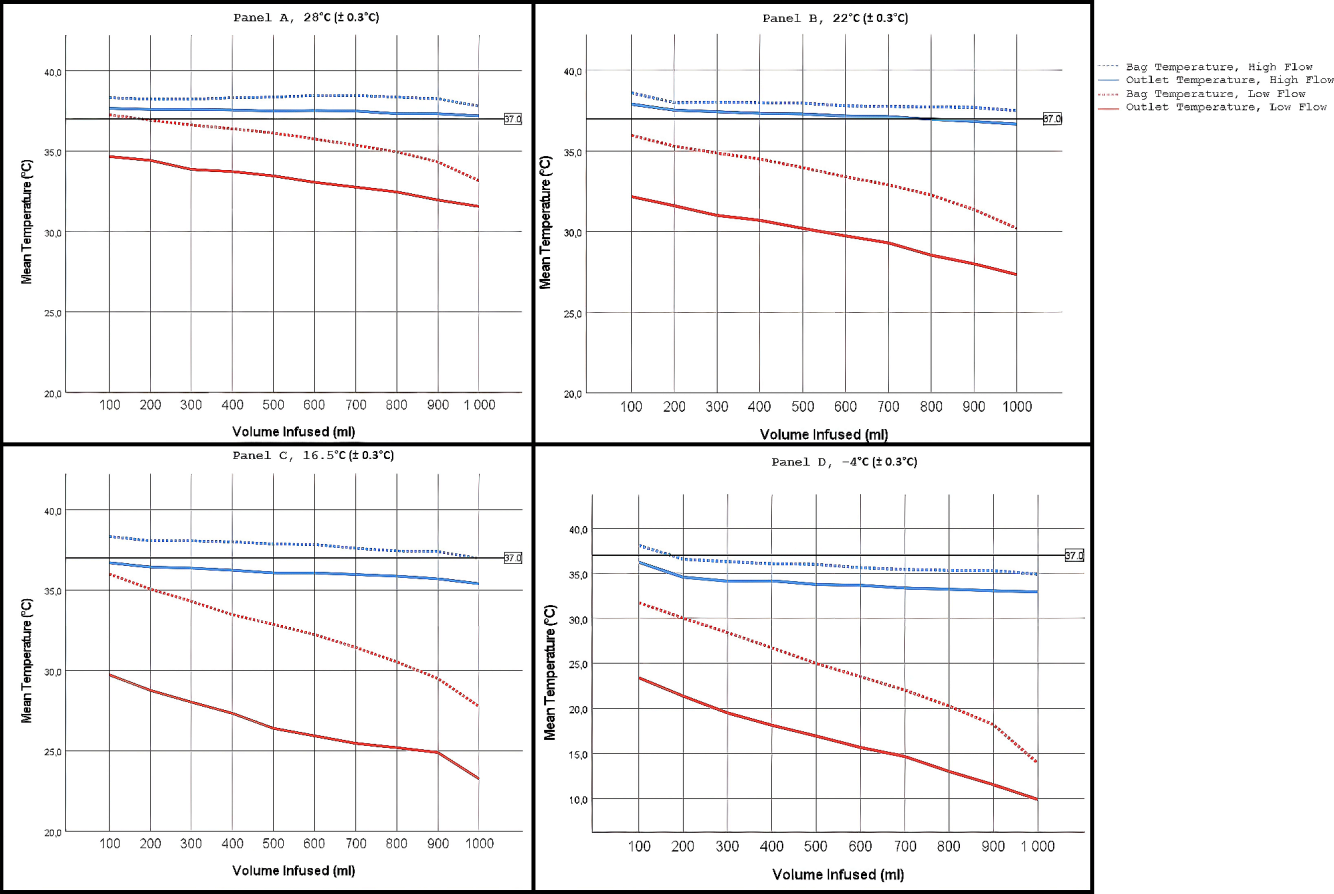
Changes in fluid temperature in infusion bags and infusion line during an infusion at different ambient temperatures

The temperature was lower in the infusion set than in the infusion bag, and the fluid temperature was affected by both the ambient temperature and the flow rate. A lower ambient temperature was associated with a greater temperature loss between the infusion bag and infusion set, with the exception of high flow at an ambient temperature of 22 °C. A low flow was associated with a greater temperature loss than a high flow (Table 2; Fig. 2).

**Table 2:** Avg. temperature loss (SD) between the infusion bag and infusion line at each 100 ml

Ambient Temperature	High Flow	Low Flow
28 °C	–0.8 °C (0.4 °C)	–2.5 °C (0.5 °C)
22 °C	–0.7 °C (0.3 °C)	–3.6 °C (0.6 °C)
16.5 °C	–1.7 °C (0.2 °C)	–5.8 °C (0.8 °C)
–4 °C	–2.1 °C (0.7 °C)	–7.6 °C (1.5 °C)

With a high flow, at an ambient temperature of 28 °C, the temperature in the infusion line remained above 37 °C throughout the infusion of 1000 ml. At an ambient temperature of 22 °C, the temperature in the infusion line was above 37 °C until approximately 700 ml had been infused. At an ambient temperature of 16.5 °C, the temperature in the infusion line was below 37 °C throughout the infusion regardless of flow rate. At an ambient temperature of − 4 °C, the temperature in the infusion line had dropped to below 35 °C after 200 ml.

With a low flow, at an ambient temperature of 28 °C, the temperature in the infusion line was below 37 °C at the first 100 ml measurement and was further reduced at each measurement. With all other measured ambient temperatures, the temperature in the infusion line never remained above 37 °C. At an ambient temperature of − 4 °C, the temperature in the infusion line was below 25 °C after 100 ml of infusion.

## Discussion

The main findings of this study are that the temperature of the fluid that is thought to replace the loss of blood in trauma is affected by the ambient temperature and flow rate, initially in the infusion bag and later in the infusion set. Lower ambient temperatures and lower flow rates are associated with a higher temperature loss. Thus, the lower the ambient temperature, the higher the flow rate needs to be to limit the temperature loss of the fluid.

### Mechanism for findings

The results show that under the described conditions, both a flow rate of 6000 ml/hour and an ambient temperature of 28 °C are needed to administer an entire bag of 1000 ml Ringer’s acetate if the fluid reaching the patient is to remain above 37 °C. When a flow rate of 1000 ml/hour is used, the fluid reaching the patient will not remain above 37 °C even at an ambient temperature of 28 °C.

In the present study, an infusion with a flow rate of 1000 ml/hour and an ambient temperature of 22 °C dropped to 33 °C after 30 min. These results are similar to those of another study in which temperature changes in 1000-ml infusion bags of sodium chloride were measured during an infusion at different temperatures. The bags were preheated to 39 °C, and at an ambient temperature of 22 °C, they reported a temperature of 30.1–31.2 °C in the infusion bag after 30 min of infusion with a flow rate of 999 ml/hour [26].

The fluid temperature is related to catheter length as well as infusion rate, as described in one study. At an ambient temperature of 26.5 °C, fluid preheated to 41 °C remained above 37 °C only 100 cm into the infusion set at a flow rate of 2100 ml/hour. At a flow rate of 1400 ml/hour, the fluid remained above 37 °C only 25 cm into the infusion set [27]. Our findings further emphasize the need for a higher flow rate to limit temperature changes during fluid resuscitation with warm fluids through long infusion sets. Thus, one way to limit the temperature change might be to use a shorter infusion set.

### Practical implications

#### Fluid temperature

The infusion rates that we have referred to as high flow are mainly used in situations of cardiac arrest or circulatory unstable patients. It is more likely that flow rates of 1000 ml/h will be used in most prehospital situations, for example, minor trauma, such as a fractured hip. The results show that warm fluid has a high temperature change at low flow rates and that the ambient temperature is rarely high enough to counteract this heat loss; hence, we routinely affect our patients’ body temperatures [23, 24, 31]. Our interpretation is that all fluids that have a temperature below body temperature will affect the patient negatively. To counteract cold stress and maintain normal body temperature, the human body increases mitochondrial thermogenesis and uses muscle activation to generate kinetic energy through shivering to generate heat. This action increases the release of catecholamines, which further increases the sympathetic drive, resulting in an increased labor of breathing and an increased heart rate. All these processes increase oxygen consumption and thereby contribute to the development of acidosis [32–36]. Hence, the fluid that reaches the body should be at least body temperature (37 °C) to be considered warm.

One concern in regard to considering fluids “warm” is the external factors causing temperature changes that occur in both the bag and the infusion set. Few previous studies have reported the location at which the fluid temperature was measured during the infusion process. In the prehospital setting, cold fluid was a contributing factor to accidental hypothermia among trauma patients when hypothermic patients were compared with normothermic patients, and the median fluid temperature was 19.5 °C in the hypothermic group versus 22 °C in the normothermic group [12]. Another study showed that it was possible to protect the patient against further heat loss by using fluids that were preheated to between 25 and 28 °C [37]. However, fluid temperatures of 4 and 23 °C have been reported to decrease the body temperature by 1 °C and 0.5 °C, respectively, in healthy individuals during an infusion of 30 ml/kg during a 30-minute interval [31].

#### Heating methods and management

Preheated infusion fluids are affected by temperature changes as soon as they are removed from the heater. At an ambient temperature of 22 °C, healthcare providers have approximately 10 min until the fluid temperature in the infusion bag drops below 37 °C. If one considers the steps needed to prepare the infusion, the volume of warm fluid that is possible to infuse decreases even further.

There are several methods to prevent temperature loss in preheated fluids. An ambient temperature of higher than 28 °C should enable infusions with a lower flow rate than 6000 ml/hour. However, such a temperature would have a negative influence on the work environment and thus increase the risk of adverse events [18].

A more reasonable alternative that has shown to be effective is some kind of insulation against heat loss from, for example, convection and radiation [38–40], but fewer than half of the ambulance units in Sweden have equipment for this [25].

Another method might be the use of an inline heater instead of preheated fluids. Inline heaters can effectively warm room-temperature fluids (20 °C) at flow rates of up to 3000 ml/h [41] but have limitations in warming colder fluids at higher flow rates [42]. Using such heaters might be a viable option when low flow rates are recommended, or a combination of preheated fluids and an inline heater might enable warm fluids at both high and low flow rates.

#### Recommendations

Prehospital emergency services should further facilitate the use of body-temperature fluids by.

- introducing equipment for insulation,
- using combined methods of heating.

Prehospital professionals should also consider.

- that the time between taking the infusion bag out of the infusion heater and the start of infusion should be as short as possible.
- a high ambient temperature (28 °C) and high flows (6000 ml/hour) should be maintained for fluid resuscitation where insulation of the equipment is not possible.
- when using bolus doses and the whole content is not consumed, that the bag should be stored in the infusion heater between infusions or, alternatively, discarded.

Infusion bags with a smaller volume and shorter infusion sets could favor fluid treatment with warm fluids, but whether these are satisfactory alternatives to current methods should be scientifically tested before they are implemented in practice.

### Strengths and limitations

The major limitation of this research is the absence of a laboratory setting, which limits the validity of the present study. To increase the validity, a setting that enabled better control of the ambient temperature could have been used. However, in a prehospital setting, it is more difficult to control external factors than in clinical studies, since there is no normative environment in the latter. The experiment was performed indoors where the influence of convection is low. Due to the windchill factor [43], the heat loss could be more marked outdoors if an infusion bag and infusion set are exposed to wind. However, it could also be less marked if radiation from, for example, the sun affects the system.

The only notable deviation in the results is the difference in temperature between the infusion bag and the infusion set at a high flow rate when comparing ambient temperatures of 28 and 22 °C (Table 2). This deviation is not consistent with the remaining data, which show that a larger difference between the fluid and ambient temperature results in higher temperature changes from the bag through the infusion line.

The flow rates in the study varied, particularly between the beginning and end of the infusion, where the flow rates decreased. This decrease is caused by reduced driving pressure as well as the fact that fluid viscosity increases with decreasing fluid temperature. This decrease may have affected the temperature change in the fluid, but we think that a significant effect is unlikely.

The infusion rate at − 4 °C ambient temperature was much higher than the rates at higher temperatures, namely, 8654 ml/hour for high flow and 1565 ml/hour for low flow compared to ∼ 6000 ml/h and ∼ 1000 ml/h, respectively. This means that temperature changes at − 4 °C were lower than they should have been with the same flow rates as those at higher temperatures. Equipment to control the infusion rates, such as infusion pumps, would have further increased the validity of the study. However, this kind of equipment rarely exists in Swedish ambulances [25], and previous studies that have measured temperature changes related to flow rate have used relatively low flow rates, which can impact the relevance for prehospital emergency care.

### Further studies

In terms of its setting, this study can be viewed as a guide to the subject. It emphasizes the need for prehospital professionals to be aware of temperature changes during fluid treatments and adjust their methods accordingly. Further studies with more rigid control over the environment are needed to define the question more clearly. As blood products are becoming more common in the prehospital setting, similar studies should be performed on these. The subject of accidental hypothermia is multifactorial, and this study only refers to a small part of it. Further studies on how the temperature of the fluid actually affects the patient are needed to increase quality of care and patient comfort in this area.

## Conclusion

This study shows that both a high infusion rate and a high ambient temperature are needed if an infusion fluid preheated to 39 °C is to remain above 37 °C when it reaches the patient using a 175-cm-long uninsulated infusion set. It is apparent that the lower the ambient temperature, the higher the flow rate needs to be to limit temperature loss of the fluid.

## Data Availability

Datasets are available upon reasonable request by email to the authors at emil.jensen84@outlook.com.

## Abbreviations

ICU: Intensive care unit
PVC: Peripheral vein catheter
